# Increased Arterial Stiffness Is Associated With Reduced Diastolic Function in Youth With Obesity and Type 2 Diabetes

**DOI:** 10.3389/fped.2021.781496

**Published:** 2021-11-29

**Authors:** Nicolas L. Madsen, Jessica E. Haley, Ryan A. Moore, Philip R. Khoury, Elaine M. Urbina

**Affiliations:** ^1^Department of Pediatrics, University of Texas Southwestern Medical Center, Dallas, TX, United States; ^2^Department of Pediatrics, Rady Children's Hospital, San Diego, CA, United States; ^3^The Heart Institute, Cincinnati Children's Hospital Medical Center and the University of Cincinnati, Cincinnati, OH, United States

**Keywords:** arterial stiffness, diastolic dysfunction, pediatrics, obesity, T2DM

## Abstract

**Background:** Increased arterial stiffness is associated with diastolic dysfunction in adults. Data in youth are lacking, so we examined the impact of arterial stiffness on diastolic function in youth.

**Methods:** We obtained diastolic function and augmentation index, pulse wave velocity, brachial artery distensibility, and carotid stiffness on 612 youth [10–24 years, 65% female, 38% normal weight, 36% obese, and 26% with type 2 diabetes mellitus (T2DM)]. Participants were classified as compliant (C) vs. stiff (S) arteries based on seven arterial stiffness parameters [Global Stiffness Index (GSI), S = GSI > 4). Mean differences in covariates were evaluated by Student's *t*-tests. A stepwise regression analysis was performed to determine if GSI was an independent predictor of diastolic function.

**Results:** Lower diastolic function and more adverse cardiovascular disease (CVD) risk factors were present in the S group (*n* = 67) than the C group (*n* = 545) (*p* < 0.001). Covariates that were associated with diastolic dysfunction were higher GSI, male sex, higher body mass index (BMI), and systolic blood pressure (SBP) *z*-score (*R*^2^ = 0.18 to 0.25; *p* ≤ 0.05).

**Conclusion:** Adverse diastolic function is seen in youth with increased arterial stiffness independent of CVD risk factors. Interventions to improve arterial stiffness prior to clinical onset of diastolic dysfunction are needed to prevent development of heart failure.

## Introduction

Effective cardiovascular disease (CVD) prevention requires identification of risk factors prior to the onset of clinical burden. While it is commonly understood that adults with obesity or obesity-related type 2 diabetes mellitus (T2DM) are at increased risk for CVD (1), the evidence regarding the extent to which these risk factors impact the pediatric age range is not well-characterized.

Diastolic dysfunction is a risk factor-related measure of target organ damage that predicts heart failure (2, 3) and CV events in adults (4). Emerging evidence suggests that pre-clinical diastolic dysfunction [diastolic dysfunction with normal systolic function and without symptoms of heart failure (5)] exists in hypertensive adolescents (6) and youth with obesity or T2DM (7). One mechanism for the development of diastolic dysfunction may be increased afterload on the heart induced by increased arterial stiffness (8, 9). Pediatric studies show that arterial damage is associated with higher left ventricular mass (10) and with reduced systolic strain (11). We sought to determine the relationship between arterial damage and diastolic function in healthy youth and those with CV risk factors including obesity and T2DM.

## Methods

The study population consisted of 612 youth (age 10–24 years, mean 18 years, 65% female, 62% non-Caucasian, and 26% with T2DM) who participated in a study comparing cardiovascular parameters among adolescents and young adults who were lean (L), obese (O), or obese with T2DM (T). Pregnant females were excluded from the study. Investigational review board approval was obtained. Written informed consent was obtained from subjects 18 years or older and from the guardian for subjects <18 years old. Written assent was obtained for subjects <18 years old.

### CV Risk Factor Measurements

The mean of two measures of height with a calibrated stadiometer (Veeder-Rood, Elizabethtown, North Carolina) and two measures of weight with a Health-O-Meter electronic scale (Jarden Consumer Solutions, Rye, New York) were used in analyses. Body mass index (BMI) was calculated as weight (kilograms) / height (meter) (2). The mean of three resting measures of blood pressure (BP) with mercury sphygmomanometry collected after 5 min of rest according to pediatric guidelines (12) was obtained. After an overnight fast, plasma glucose was measured with a Hitachi model 704 glucose analyzer (Roche Hitachi, Indianapolis, Indiana) with intra-assay and inter-assay coefficients of variation of 1.2 and 1.6%, respectively. Plasma insulin was measured by radioimmunoassay with an anti-insulin serum raised in guinea pigs, indium125-labeled insulin (Linco, St. Louis, Missouri), and a double-antibody method to separate bound from free tracer with a sensitivity of 2 mmol (intra-assay and inter-assay coefficients of variation of 5 and 8%, respectively). Glycated hemoglobin A1c (HbA1c) was measured by use of high-pressure liquid chromatography. Fasting plasma lipid profiles were performed with standardized methods from the National Heart Lung and Blood Institute–Centers for Disease Control and Prevention, and low-density lipoprotein cholesterol concentration was calculated with the Friedewald equation. C-reactive protein (CRP) was measured with a high-sensitivity enzyme-linked immunosorbent assay.

### Arterial Stiffness Measurements

Vascular function testing was conducted after 5 min of rest in the supine position. Three measures of brachial artery distensibility (BrachD) were obtained with a DynaPulse Pathway instrument (Pulse Metric, Inc., San Diego, California). This device derives brachial artery pressure curves from arterial pressure signals obtained from a standard cuff sphygmomanometer. Brachial artery compliance is derived from waveform parameters, and then BrachD is calculated as compliance normalized to baseline brachial artery diameter (estimated from a regression equation developed from ultrasound, adjusting for sex and body size). This variable is equivalent to other measures of distensibility, such as those measured with ultrasonography, in that it represents the relative change in volume per unit of pressure and is expressed with the units of %change/mmHg. Repeat measures in our laboratory show coefficients of variability <9%.

Three measures of pulse wave velocity (PWV) were measured and averaged with a SphygmoCor SCOR-PVx System (Atcor Medical, Sydney, Australia). PWV is a measure of the difference in the carotid-to-distal path length divided by the difference in R-wave-to-waveform foot times (m/s). Specifically, electrocardiography (ECG) leads were applied to the carotid artery, the sternal notch, and the distal artery of interest (femoral, radial, and dorsalis pedis). A pressure tonometer the size of a pencil is placed on the proximal artery (carotid) then distal to obtain arterial waveforms gated to the R-wave on the electrocardiography tracing. The ECG recording was used to measure heart rate. Repeat measures in our laboratory show coefficients of variability <7%.

Three measures of augmentation index (AIx) were collected with the SphygmoCor device. The pressure sensor is applied to the radial artery to collect radial artery pressure waves that are calibrated to a non-invasive blood pressure (Pulse Metric, Inc., San Diego, California). A generalized transfer function validated against invasive catheterization data is used to calculate central (aortic) systolic blood pressure (SBP), diastolic blood pressure (DBP), mean arterial pressure (MAP), and pulse pressure (PP) and reconstruct the central aortic pressure curve. AIx, adjusted to a heart rate of 75 beats per minute, is calculated utilizing the ascending aorta pressure curve. AIx is the pressure difference between the primary (main outgoing wave) and the reflected wave of the central arterial waveform, expressed as a percentage of the central pulse pressure. Reproducibility studies in our laboratory demonstrated intra-class correlation coefficients between 0.7 and 0.9 for all variables.

### Carotid Ultrasonography

Carotid ultrasound studies were performed by a single registered vascular technologist using high-resolution B-mode ultrasonography (GE Vivid 7; GE Healthcare, Milwaukee, Wisconsin) with a high-resolution linear array vascular transducer (7.5 MHz). An optimal two-dimensional (2D) image of the common carotid artery was obtained, where both the near and far wall intima/media complex were well-visualized. The M-mode cursor was then placed 1 cm proximal to the beginning of the carotid artery bulb. Multiple image loops were digitally transmitted by use of the Camtronics Medical System (Camtronics Medical Systems, Hartland, Wisconsin) for off-line reading. The maximal and minimal lumen diameters were read from the M-mode tracing for calculations of carotid stiffness. Calculations included arterial compliance (AC), beta stiffness index (β), circumferential arterial strain (CAS), Peterson's elastic modulus (PEM), and Young's elastic modulus (YEM). Because of pulse-wave amplification along the arterial tree, which results in overestimation of brachial SBP, the central BP calculated from the radial artery pressure curve using the SphygmoCor device (obtained no more than 30 min before the carotid scan) was used in the calculations of carotid artery stiffness.

### Echocardiographic Technique

Echocardiograms were obtained with a GE Vivid 5 or 7 (Milwaukee, WI, USA) or Philips Sonos 5500 (Andover, MA, USA) ultrasound system. A complete 2D pulsed Doppler, tissue Doppler, and color Doppler echocardiographic examination was performed on each participant. All images were obtained with the participant in the left lateral decubitus position to acquire parasternal long-axis, parasternal short-axis, and apical four-chamber views for a total of three cardiac cycles. Left atrial diameter (LAD) was measured in the long axis and indexed to height (LAD/ht). Measurement was performed off-line by either of two sonographers using a Cardiology Analysis System (Digisonics, Houston, TX, USA).

The assessment of mitral inflow velocity was obtained with pulsed wave Doppler parallel to mitral inflow in the apical four-chamber view, and maximal velocity measured at the mitral valve leaflet tips. The mitral peak *E* (early filling) and *A* (inflow with atrial contraction) waves were measured off-line, and an *E*/*A* ratio was calculated. Tissue Doppler imaging of myocardial flow velocities was acquired in the apical four-chamber view. The peak and late velocities of mitral annular flow were recorded at both the septal annulus (*e*′-sept, *a*′-sept) and lateral annulus (*e*′-lat, *a*′-lat). The *e*′/*a*′ ratios were calculated in addition to *E*/*e*′-lat and *E*/*e*′-sept ratios. The *E*/*e*′ ratio corrects for myocardial relaxation in transmitral flow (*E*) and has been shown to correlate with left ventricular (LV) end-diastolic pressure (7). In adults, an *E*/*e*′-lat of >10 is predictive of elevated LV filling pressures, and <6 is normal. In addition, the left atrial size was assessed by two-dimensional-directed M-mode and indexed to height.

### Statistical Analysis

All analyses were performed with Statistical Analysis Software (SAS®, version 9.1.3, Cary, North Carolina). Variance-stabilizing measures to transform non-normal values were performed as needed. The 95th percentile for each of the seven arterial stiffness measures (BrachD, AIx, PWV, AC, β, CAS, PEM, and YEM) for lean subjects without diabetes was determined. Subjects were given a score of 1 for the parameter if ≥95th percentile for the lean group (≤ 5th percentile for AC and BrachD) and 0 if below the cutpoint (overall, a total of 7 points are possible). Global Stiffness Index (GSI) was calculated as the sum of the stiffness points for each of the four measures of carotid artery stiffness and the three non-ultrasound measures of arterial stiffness. The GSI has been shown to be linearly related to LV mass index in a previous study (10). Subjects were stratified into either “compliant arteries” (CA) or “stiff arteries” (SA) based on their GSI score (a score of 4 or greater, which was the 95th percentile for GSI for the lean, healthy group, qualified as stiff). Average values for demographic, anthropometric, BP, and laboratory values were obtained for each group. Student's *t*-tests were performed to determine differences by stiffness classification. The χ^2^ analyses were performed for categorical variables. Bivariate correlations were calculated for GSI, covariates, and diastolic function variables. Variables that were significant in the bivariate analysis were included as potential independent predictors in the general linear model analyses. The full model contained the following data: demographic (age, race/ethnicity, sex, and presence of T2DM), anthropometric (BMI *z*-score), hemodynamic (SBP *z*-score, DBP *z*-score, and HR), and laboratory (fasting glucose, fasting insulin, HbA1c, low-density lipoprotein cholesterol, high-density lipoprotein cholesterol, triglycerides, and CRP). The significance of each covariate in the initial model was assessed, and non-significant terms were removed by backward elimination until all remaining covariates or their interaction terms were significant. Robustness of the models was assessed with the use of the maximum *R*-square technique.

## Results

The population included 612 youth (10–24 years, 65% female, and 62% non-Caucasian) enrolled in one of three groups (38% lean, 36% obese without T2DM, and 26% obese with T2DM). When stratified as having compliant or stiff arteries (Table 1), participants with stiff arteries were older and more obese and had higher peripheral BP and heart rate, a more adverse lipid and metabolic profile, and more evidence of inflammation (all *p* ≤ 0.01). Specifically, lipids in the stiff group were within normal limits, but glucose, insulin, and HbA1c were elevated, since more diabetics were included in the group. Additionally, the stiff group had a mean SBP in the elevated BP category according to BP guidelines (12).

**Table 1:** Demographics and metabolic profile of study participants stratified by Global Stiffness Index category (*n* = 612, mean ± SD or frequency).

**Variable**	**Compliant (*n* = 545)**	**Stiff (*n* = 67)**	***P* value**
Age (years)	17.8 ± 3.3	19.5 ± 3.2	<0.01
Sex (% male)	192 (35.2%)	18 (26.9%)	NA
Race (% non-Caucasian)	335 (61.5%)	46 (68.6%)	NA
Presence of T2DM (%)	127 (23.3%)	30 (44.8%)	NA
Weight (kg)	87 ± 31	113 ± 26	<0.01
Height (cm)	167 ± 11	168 ± 10	NS
BMI (kg/m^2^)	31 ± 10	40 ± 8	<0.01
SBP (mmHg)	114 ± 12	124 ± 12	<0.01
DBP (mmHg)	63 ± 12	71 ± 15	<0.01
HR (beats/min)	66 ± 11	72 ± 11	<0.01
TChol (mg/dl)	169 ± 35	184 ± 43	<0.01
LDL-C (mg/dl)	99 ± 29	114 ± 40	<0.01
HDL-C (mg/dl)	50 ± 13	46 ± 11	<0.01
TG (mg/dl)	96 ± 67	126 ± 73	<0.01
Fasting glucose (mg/dl)	103 ± 43	123 ± 70	<0.01
Fasting insulin (mU/ml)	18 ± 15	23 ± 12	<0.01
HbA1c (%)	5.97 ± 1.8	6.72 ± 2.3	<0.01
hsCRP (mg/l)	4 ± 6.5	6.5 ± 7.3	<0.01

*T2DM = type 2 diabetes mellitus; BMI = body mass index; SBP = systolic blood pressure; DBP = diastolic blood pressure; HR = heart rate; TChol = total cholesterol; LDL-C = low density lipoprotein concentration; HDL-C = high density lipoprotein concentration; HbA1c = glycoselated hemoglobin; hsCRP = high sensitivity C-reactive protein*.

**Figure 1:**
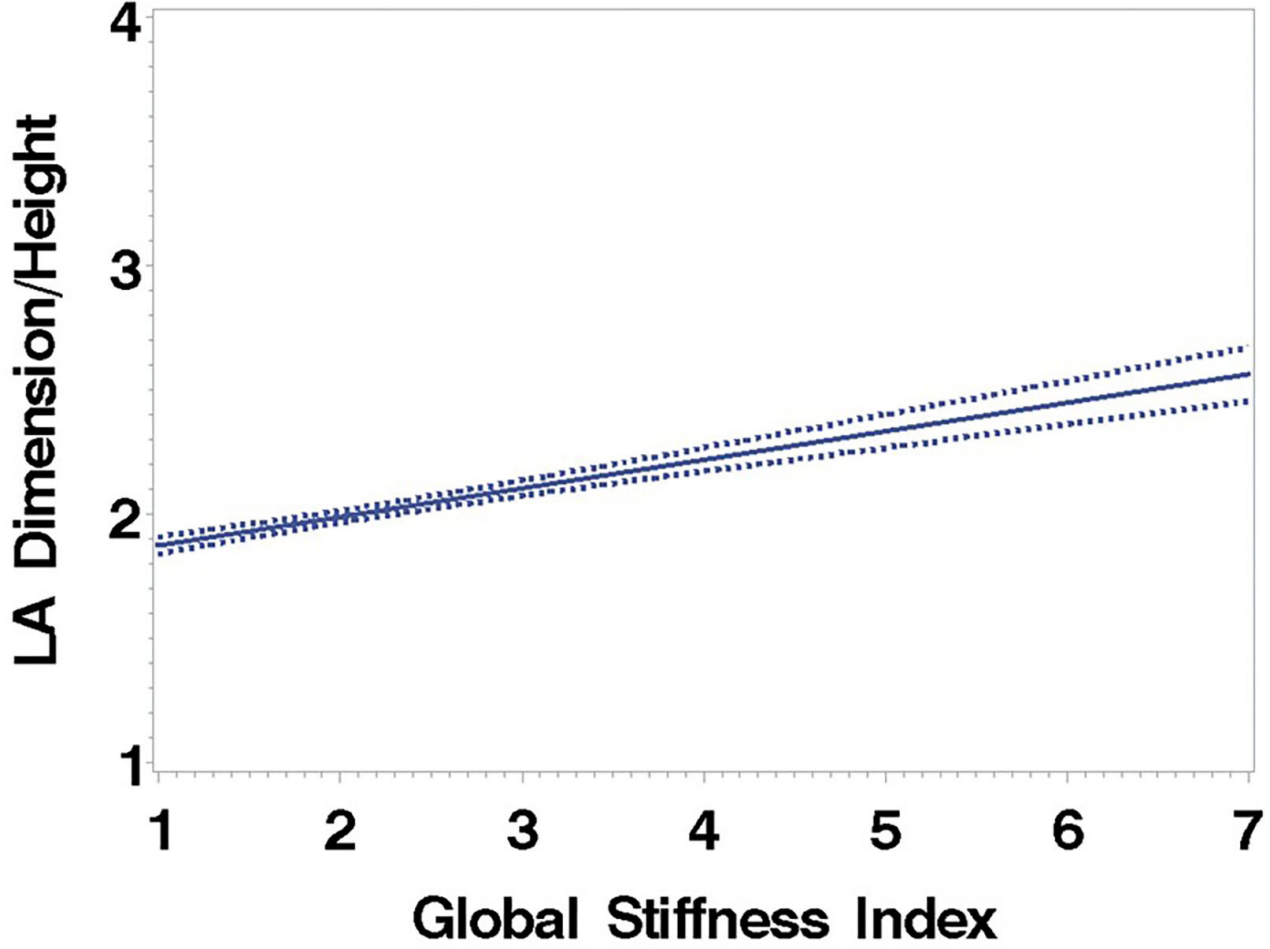
Left atrial dimension/height regressed on the Global Stiffness Index (mean with 95% confidence limit). *R*^2^ = 0.40; *P* for slope differs from zero <0.0001 in fully adjusted model.

**Figure 2:**
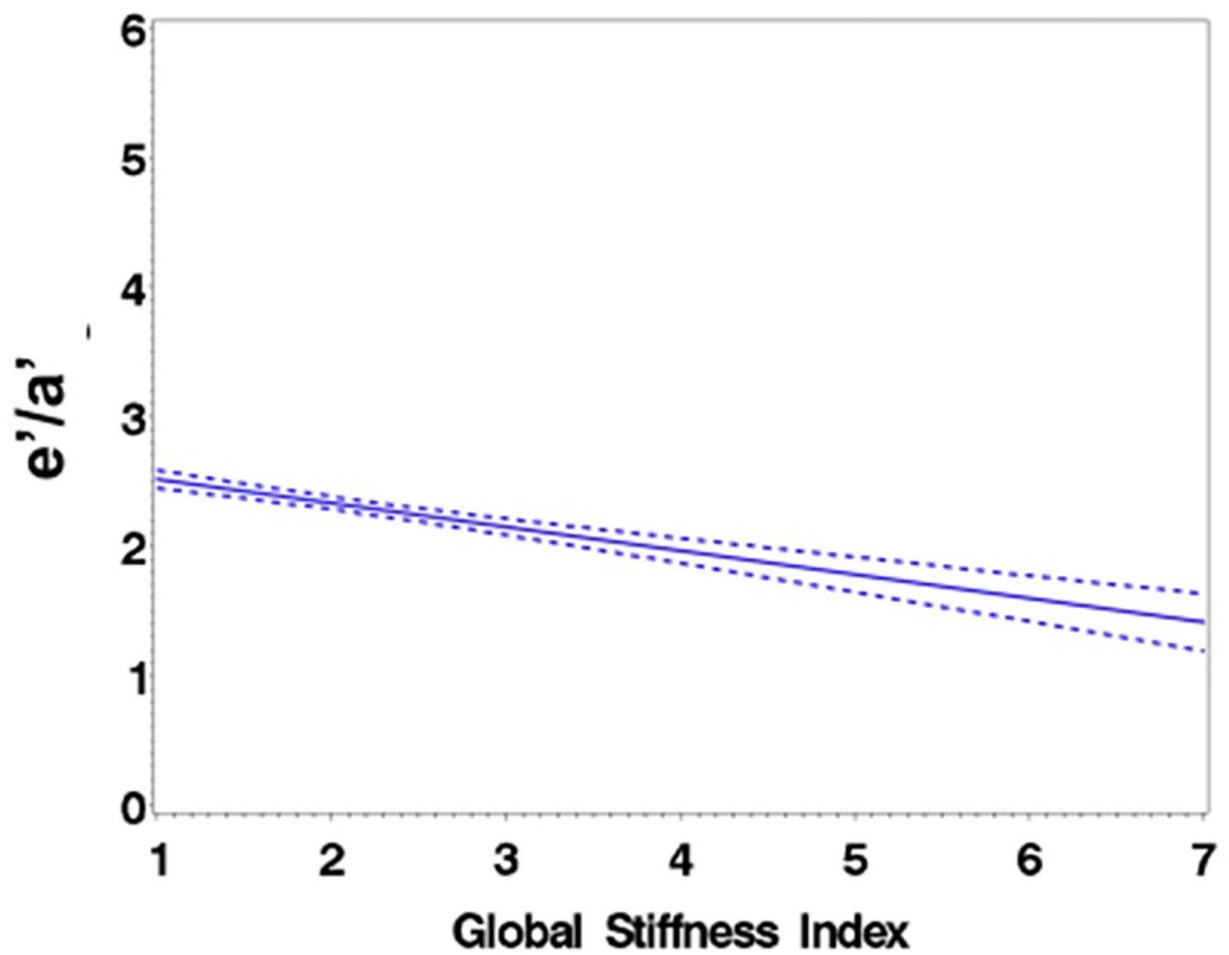
Regression of *E*/*A* ratio on the Global Stiffness Index (mean with 95% confidence limit). *R*^2^ = 0.16; *P* for slope differs from zero <0.0001 in fully adjusted model.

**Figure 3:**
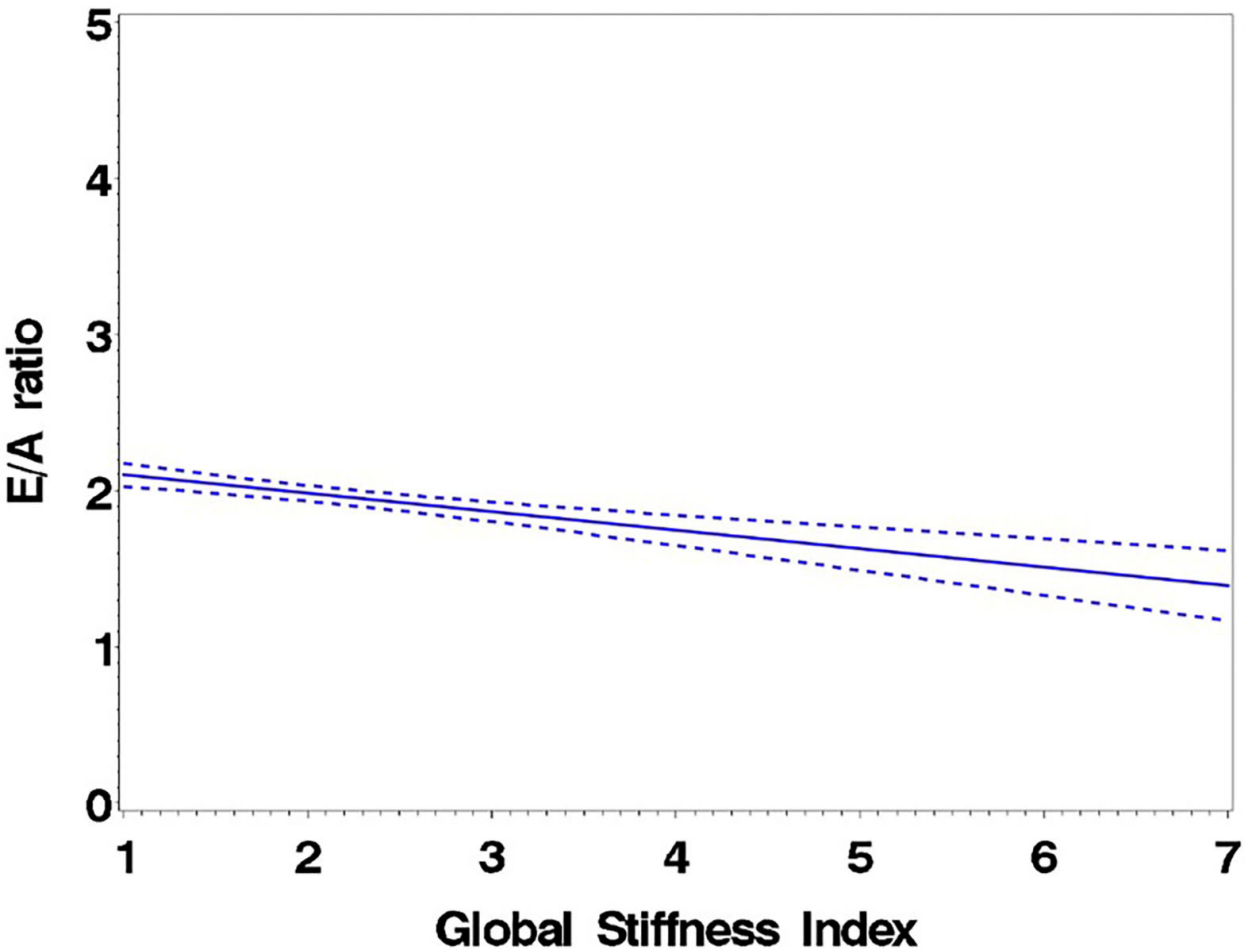
Regression of *e*′/*a*′ average on the Global Stiffness Index (mean with 95% confidence limit). *R*^2^ = 0.29; *P* for slope differs from zero <0.0001.

Consistent with stratification by GSI, the stiff cohort had arterial function measures (AIx, PWV, β, PEM, YEM, AC, CAS, and BrachD) that were in the direction of higher arterial stiffness (Table 2). The stiff group also had significantly lower *E*/*A* ratio and *e*′/*a*′, as well as higher *E*/*e*′, all suggesting pre-clinical diastolic dysfunction (Table 2). There was a linear relationship between increasing levels of GSI (0–7) and lower diastolic function including increased LAD/ht (Figure 1) and between lower *E*/*A* ratio (Figure 2) and *e*′/*a*′ (Figure 3). There was a similar increase in *E*/*e*′ across GSI score (data not shown).

**Table 2:** Cardiovascular parameters stratified by arterial stiffness category (mean ± SD).

**Variables**	**Compliant (*n* = 545)**	**Stiff (*n* = 67)**	***P* value**
**Stiffness variables (means)**			
AIx (%)	1.65 ± 11	8.27 ± 13	<0.01
BrachD (%Δ/mmHg)	6.15 ± 1.3	4.73 ± 0.6	<0.01
PWV (m/s)	5.9 ± 1	7.3 ± 1.2	<0.01
AC (mm/mmHg)	0.27 ± 0.07	0.20 ± 0.06	<0.01
Beta (unitless)	2.2 ± 0.5	2.9 ± 0.9	<0.01
CAS (unitless)	0.19 ± 0.04	0.16 ± 0.05	<0.01
PEM (mmHg)	192 ± 65	209 ± 77	NS
YEM (mmHg/mm)	256 ± 112	403 ± 150	<0.01
**Diastolic variables**			
*E*/*A* ratio	1.99 ± 0.55	1.76 ± 0.43	<0.01
*e′*/*a′* avg	2.36 ± 0.65	1.91 ± 0.48	<0.01
*E*/*e′* avg (LVEDP)	6.47 ± 1.43	7.29 ± 1.68	<0.01
LA diameter/ht (cm)	1.98 ± 0.32	2.21 ± 0.33	<0.01

General linear models demonstrated that GSI was independently related to diastolic function (*p* ≤ 0.0001 for LAD/ht, *E*/*A*, and *e*′/*a*′). Other important covariates associated with lower diastolic function were male sex, higher BMI and SBP *z*-score, age, LDL, CRP, and HR (*R*^2^ = 0.16 to 0.40; model *p* ≤ 0.001 and all parameters *p* ≤ 0.05) (Table 3).

**Table 3:** Independent determinants of diastolic function.

**Variable**	**LAD/ht**	***E*/*A***	*e^**′**^*/a^′^^*^	*E*/e^′^^*^
Intercept	1.73	1.48	1.73	1.67
Presence of T2MD		−0.079		0.072
GSI	0.029	−0.049	−0.032	NS
Male sex			0.058	
BMI *z*-score		−0.029	−0.051	0.060
Age (years)		−0.013	−0.017	
SBP *z*-score				0.072
HR (beats/min)		−0.0067	−0.0075	
LDL (mg/dl)			−0.00075	
CRP (mg/dl)	0.0052			
***R^2^***	**0.40**	**0.16**	**0.29**	**0.23**

*All models p ≤ 0.0001 and all parameters p ≤ 0.05*.

* *Average of septal and lateral TDI velocities*. *All measures of diastolic function are unitless ratios*.

## Discussion

Our study findings demonstrate that higher arterial stiffness, independent of traditional CVD risk factors, is associated with lower diastolic function in youth. Importantly, these changes in youth are pre-clinical and represent an early form of cardiac disease that is measurable before most other traditional determinants of cardiac disease, such as overt heart failure.

Clinical symptoms of heart failure are common in the adult population, experienced by at least 6.2 million Americans according to American Heart Association data (1). However, many more adults may have asymptomatic diastolic dysfunction, with the prevalence in the Framingham Heart Study reported to be 36% (1). This is relevant since diastolic dysfunction is predictive of incident heart failure (3), reduced quality of life (13), and all-cause mortality (3, 5, 14).

Adult studies have demonstrated that increased pulse pressure (a crude surrogate for arterial stiffness) is independently predictive of not only diastolic dysfunction (15–17) but also heart failure with preserved ejection fraction (18, 19). In addition, carotid artery wall stiffness (20, 21) and aortic compliance, a similar parameter, positively correlate with LV diastolic function (22). Similar to our results, increased PWV is independently associated with diastolic dysfunction in patients with hypertension (23), type 2 diabetes (24), clustered CV risk factors (25), and suspected coronary artery disease (26, 27). Measures of wave reflection including augmentation index are also associated with diastolic dysfunction (28) and LV filling pressure (*E*/*e*′) (9). Although an association cannot prove causality, investigators have proposed that as cardiac output falls with worsening diastolic function, neurohumoral activation, and vasoconstriction increase vessel tone to maintain mean arterial pressure and thereby increase vascular smooth muscle mass, tone, and fibrosis, resulting in increased stiffness (29). A direct relationship between neurohumoral activation and increased carotid stiffness has been demonstrated in subjects with heart failure (30). It is also possible that increased pulse wave velocity generates an earlier reflected wave in the cardiac cycle, increasing late systolic afterload, affecting thick–thin myofilament interactions and crossbridge dissociation, and leading to impaired relaxation (31, 32). The importance of increased arterial stiffness in determining diastolic function is seen in studies of normo- and hypertensive adults, where relaxation assessed with tissue Doppler varies inversely with afterload and vascular stiffness (31). Measurement of ventricular–arterial coupling (VAC = ratio of arterial elastance to end-systolic elastance) is also finding increasing usage as VAC predicts outcomes in adults with cardiac disease and heart failure (33). Our study provides a different method to evaluate the relationship between arterial and cardiac function.

Few data are available examining the relationship between arterial stiffness and diastolic function in adolescents. Our previous work demonstrated that increased left ventricular mass was associated with higher arterial stiffness (9), and carotid intima media thickness was associated with reduced systolic strain in healthy youth and those with obesity and T2DM (11). One small study found a relationship between left atrial strain (reflecting diastolic dysfunction) and measures of insulin resistance in obese children (34). Bradley et al. found both increased arterial stiffness and diastolic dysfunction in a group of adolescents with type 1 diabetes mellitus, but did not evaluate the association between the two factors (35). In a later study of children with type 1 diabetes, endothelial function (brachial flow-mediated dilation), which is associated with arterial stiffness, was inversely correlated with isovolumic relaxation time, another echocardiographic measure of diastolic function (36). Arterial stiffness has also been associated with elevated LVM in youth after repair of coarctation (37–39). Altered wave reflections leading to increased afterload on the heart has been proposed as a mechanism explaining this observation in youth with a history of coarctation repaired at a young age (40).

Adult studies have also examined the impact of metabolic syndrome (41) and T2DM (42) on arterial stiffness and diastolic function. Roes et al. (41) used MRI to evaluate diastolic dysfunction and found increased PWV and impaired LV diastolic function in subjects with metabolic syndrome, regardless of blood pressure. However, the relationship between PWV and diastolic dysfunction was not examined. Sharman et al. (1) found central pulse pressure, reflecting central arterial stiffness similar to PWV, but not brachial pulse pressure, reflecting stiffness of medium muscular artery, independently predicted diastolic dysfunction in subjects with T2DM. They concluded that increased central stiffness, possibly due to amplified pressure wave reflections, was one potential etiology of the observed abnormalities in LV diastolic function in patients with T2DM. Our work extends the observations of a relationship between arterial aging and diastolic function to youth who are healthy, have uncomplicated obesity, or have obesity-related T2DM.

Many studies have employed exercise interventions to improve arterial parameters. Adult studies have shown a positive association between exercise training and improvement in endothelial dysfunction in adults with both insulin resistance (43, 44) and T2DM (44, 45). The study by Okada et al. actually saw a decreased rate of cardiovascular events in those participating in the exercise program (45). Similarly, exercise training has been found to improve endothelial function (as measured by FMD) in adolescents with obesity (46, 47) and T2DM (48). Some studies have attempted to reverse cardiac dysfunction, with a few demonstrating improved left ventricular diastolic function in obese adults following successful weight loss (49, 50). The effect of lifestyle modification on diastolic function has not been studied extensively in youth. However, the above findings suggest that the implementation of an exercise program in obese and diabetic patients may be an appropriate investment of health care dollars to decrease future risk of cardiovascular disease.

## Limitations

Our cross-sectional design does not allow us to determine the time sequence for the development of changes in arterial stiffness and cardiac diastolic function. As a result, we cannot speculate about causality and cannot precisely determine whether increased arterial stiffness preceded the development of diastolic dysfunction or if the reverse is true. In addition, we do not know if they developed simultaneously.

Because of the original study design, our population contains a large proportion of obese subjects and subjects with T2DM that may limit the generalizability of our findings to other populations. Furthermore, both adiposity and the presence of T2DM were important determinants of diastolic function. We were neither able to assess the duration of obesity nor is the duration of T2DM certain, as the earliest phase may be asymptomatic and go unrecognized.

There may also have been other non-measured confounders (for example, activity pattern and fitness level) that affected the vascular-cardiac relationship. However, our findings are similar to results obtained in adults with known cardiovascular risk factors. Finally, equipment and expertise in collecting ultrasound measures of carotid artery stiffness and non-ultrasound measures of arterial stiffness may not be readily available to many pediatric care providers, thus limiting the applicability of the GSI calculation to the clinical setting.

## Conclusions

We conclude that lower diastolic function is seen in youth with increased arterial stiffness independent of traditional CVD risk factors. Arterial stiffness likely contributes to reduction in diastolic function by increased pulse pressure and LV afterload. Screening for arterial stiffness and diastolic dysfunction in obese or T2DM adolescents may identify youth at increased risk for developing early CVD and provide the temporal opportunity for normalization of pre-clinical disease.

## Data Availability Statement

The raw data supporting the conclusions of this article will be made available by the authors, without undue reservation.

## Ethics Statement

The studies involving human participants were reviewed and approved by IRB committee, Cincinnati Children's Hospital Medical Center. Written informed consent to participate in this study was provided by the participants' legal guardian/next of kin.

## Author Contributions

PK and EU designed the study, collected the data, performed data analyses, and contributed to the manuscript. NM, JH, and RM created the initial draft of the manuscript and contributed to final edits. All authors contributed to the article and approved the submitted version.

## Funding

This study was supported by the National Institutes of Health (National Heart, Lung, and Blood Institute) grants R01 HL076269 (CV Disease in Adolescents With Type 2 Diabetes) and R01 HL105591 (CV Aging Study) and partially funded by the National Center for Research Resources and the National Center for Advancing Translational Sciences, National Institutes of Health, through grant 8 UL1 TR001425.

## Author Disclaimer

The content is solely the responsibility of the authors and does not necessarily represent the official views of the NIH.

## Conflict of Interest

EU received grant funding for this project from NIH (NHLBI) R01 HL105591. The remaining authors declare that the research was conducted in the absence of any commercial or financial relationships that could be construed as a potential conflict of interest.

## Publisher's Note

All claims expressed in this article are solely those of the authors and do not necessarily represent those of their affiliated organizations, or those of the publisher, the editors and the reviewers. Any product that may be evaluated in this article, or claim that may be made by its manufacturer, is not guaranteed or endorsed by the publisher.
